# Syntactic–Semantic Detection of Clone-Caused Vulnerabilities in the IoT Devices

**DOI:** 10.3390/s24227251

**Published:** 2024-11-13

**Authors:** Maxim Kalinin, Nikita Gribkov

**Affiliations:** Institute of Computer Science and Cybersecurity, Peter the Great St. Petersburg Polytechnic University, 29 Polytekhnicheskaya ul., 195251 St. Petersburg, Russia; gribkov@ibks.spbstu.ru

**Keywords:** attributed abstract syntax tree, code clone, detection, graph neural network, IoT software, semantic analysis, Siamese network, similarity, syntactic analysis, vulnerability

## Abstract

This paper addresses the problem of IoT security caused by code cloning when developing a massive variety of different smart devices. A clone detection method is proposed to identify clone-caused vulnerabilities in IoT software. A hybrid solution combines syntactic and semantic analyses of the code. Based on the recovered code, an attributed abstract syntax tree is constructed for each code fragment. All nodes of the commonly used abstract syntax tree are proposed to be weighted with semantic attribute vectors. Each attributed tree is then encoded as a semantic vector using a Deep Graph Neural Network. Two graph networks are combined into a Siamese neural model, allowing training to generate semantic vectors and compare vector pairs within each training epoch. Semantic analysis is also applied to clones with low similarity metric values. This allows one to correct the similarity decision in the case of incorrect matching of functions at the syntactic level. To automate the search for clones, the BinDiff algorithm is added in the first stage to accurately select clone candidates. This has a positive impact on the ability to apply the proposed method to large sets of binary code. In an experimental study, the developed method—compared to BinDiff, Gemini, and Asteria tools—has demonstrated the highest efficiency.

## 1. Introduction

In the last decade, the number of Internet of Things (IoT) devices has grown rapidly. According to the IoT Analytics’ State of IoT Summer 2024 report [1], there were 16.6 billion connected IoT devices by the end of 2023. They expect this number to grow to 18.8 billion by the end of 2024 and forecast 41.1 billion devices by 2030. Several use cases have emerged from the IoT, changing traditional business models, such as replacing in-person healthcare with virtual and remote monitoring, improving power planting by smart sensors and adaptive delivery, etc. Gartner estimates that the IoT in key industries reached over USD 268 billion.

In 2022, and IoT devices are forecasted to grow at a compound annual growth rate (CAGR) of 15% from 2021 to 2025 [2].

In such conditions, code cloning is widely used by IoT developers to improve production efficiency. IoT device software often uses code cloning and relies heavily on software reuse. Unlike traditional computers, IoT devices are more diverse. IoT software cloning can occur in several scenarios:

- Using proprietary program code from a corporate code database when developing commercial software;
- Using open-source code repositories when developing proprietary software;
- Using open-source code repositories when developing open-source software.

Considering two different IoT devices, if the open software used by the first one has mature and ready-to-use code, then the developer of the second device can quickly create a customized software version based on the first device by modifying the available code (e.g., drivers, file systems, and operating system modules). At the same time, any vulnerabilities taking place in the original device can also be transferred to the second device. These vulnerabilities are defined as clone-caused vulnerabilities [3]. For IoT production and sales, security often comes second, and although the open source code can be patched after vulnerability disclosure, its clones are rarely updated or even monitored after production, leading to a large number of security breaches [4]. For example, the Debian operating system for IoT devices has confirmed the existence of 145 unpatched clone-caused vulnerabilities [5].

Apart from this, software code can be cloned from different architectures to IoT environments. As a result, code-caused vulnerabilities are also being ported to the IoT software from different platforms. Attackers who master the exploitation of a specific vulnerability can use it to attack IoT devices on different platforms, which is highly dangerous and may lead to massive crashes in the IoT.

Code clone searching can also be useful in other use cases. If a vulnerability is found in an IoT device, code clone detection can help one to check other versions of the reused software for the presence of a similar defect. Also, comparing different versions of software of the same product allows identifying security patches used to close the vulnerabilities. This capability can also be used to verify the security of the same versions of IoT devices to detect undeclared insertions and assess the risks of using them. Code clone detection can be mighty, making evident the illegal reproduction of proprietary programs in IoT device replicas by unfair developers.

**Figure 1:**
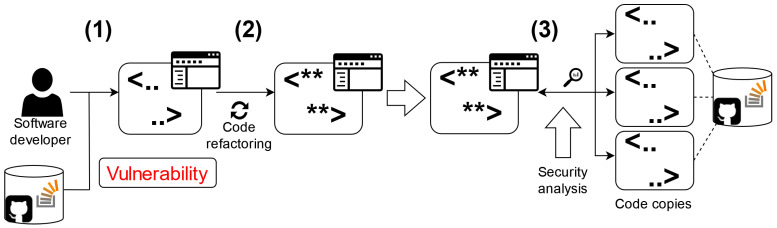
Code clone detection scenario: clone-caused vulnerability detection.

Figure 1 demonstrates one of the possible scenarios for using code clone detection. In stage 1, a developer creates IoT device software using an available code database (open repository, Internet resource, code library, etc.), or, in the case of proprietary development, a corporate code base. The original source may contain an IoT software vulnerability. In the process of development, the code is refactored. It is modified for adaptation to target use conditions (stage 2). The functional abilities of the cloned code do not change, and the clone-caused vulnerability stays in the code. Then, a binary is built. While making it, the debug symbols are lost, and the grammar of the binary code replaces the grammar of the source code. In this stage, the code syntax changes, but its semantics remain the same. During the execution of the binary, a clone-caused vulnerability may run in the IoT device. Special security analysis is required to detect this vulnerability. Security analysts utilize special detection tools to search for and classify similar code fragments containing clone-caused vulnerabilities (stage 3).

To address the IoT security problem caused by code cloning, various methods have been proposed since the initial research on clone-caused vulnerabilities in 2007 [6]. These methods operate at different granularity levels and implement various mechanisms, e.g., intelligent binary signature similarity measurements, abstract syntax trees (ASTs), control flow graphs (CFGs), program dependence graphs (PDGs), and machine learning methods [7,8,9,10,11,12]. Although these techniques achieved some success in code clone detection, they are limited by two barriers [13]:

- Incomplete and inaccurate types of semantic information are captured from functions, leading to a high rate of false positives. For example, the BinGo tool [14] relies on the CFG to generate a function signature model. However, CFGs are significantly divergent across different platforms, resulting in BinGo’s cross-platform code clone detection accuracy being less than 60%;
- Most methods require substantial data processing power, making them challenging to apply to complex tasks. For example, the Genius tool [12] uses spectral algorithms for clustering and graph matching. The Gemini tool [7] applies a deep learning model to process CFGs, and, consequently, it loses a large portion of semantic information while optimizing the data mining procedure. Most detectors work on a syntactic level of clone searching.

To overcome these problems, a hybrid solution is presented in this paper that combines syntactic and semantic analyses to detect the code clones. The following are key contributions of the presented research:

- A formal description of the code clone search is proposed. On this basis, a hybrid method for code clone detection is proposed that combines syntactic and semantic analyses. This method utilizes an attributed abstract syntax tree, our improvement of the commonly used abstract syntax tree that was extended with a vector representation of code features, and a Siamese network of two deep graph neural networks. Therefore, the proposed method combines low-level code feature processing and high-level semantic analysis;
- An experimental study of the proposed method was conducted, demonstrating its efficiency in maintaining IoT software. It shows better output quality (e.g., AUC 0.962) than the tested competitors—BinDiff, Gemini, and Asteria utilities.

In practice, the proposed method can be integrated into the production chain as a preliminary stage before the commissioning of IoT products: the software of devices should be specially checked for vulnerabilities and undeclared capabilities, especially if the products are developed for critical infrastructures.

The rest of this paper is organized as follows: Section 2 presents the related works and our hybrid syntactic–semantic method for code clone detection; Section 3 shows the results of an experimental study, demonstrating the efficiency of the developed method; Section 4 summarizes our achievements; and, finally, Section 5 concludes this research and presents further plans.

## 2. Materials and Methods

### 2.1. Related Works

A code sample is recognized as a clone if it satisfies several given definitions of similarity [15]. Currently, four types of code clones are recognized:

1. Exact clones: the program code is re-used as is without any modifications.
2. Renamed clones: syntactically identical clones. Variables, types, spaces, layout, and comments can be modified.
3. Restructured clones: this is based on renamed clones, and code fragments can be re-edited by adding, removing, or modifying the statements.
4. Semantic clones: two code samples differ in syntax, but implement the same function and, thus, have the same semantics.

Most up-to-date solutions are focused on the first three types of code clones (e.g., [4,8,9,10,11]). For example, CVdetector [16] traverses the grammar of vulnerable code fragments, constructs a vulnerability feature matrix and a feature vector for key nodes using an analysis tree, and detects various types of vulnerable code by applying clustering. This method struggles with a large amount of code. VulPecker [17] combines ASTs, PDGs, and other code feature sources, extracts code features according to their type, selects a corresponding algorithm for similarity comparison, and detects the re-used code by applying a support vector machine (SVM). The VUDDY utility [18] generated fingerprints at the function level, enabling the detection of exact and renamed clones.

VulDeePecker [3] is a state-of-the-art tool that first used deep machine learning to extract code features and detect the restructured clones, but it can only handle API. Following this method, a series of modifications were proposed, inspired by different machine learning models, e.g., convolutional neural networks, language models, and multi-classifiers [19,20,21,22,23]. This range of methods focuses on code abstraction and the extraction of code features through syntactic analysis. However, more accurate clone detection requires additional information, which can be found at a higher level of code semantics.

**Figure 2:**
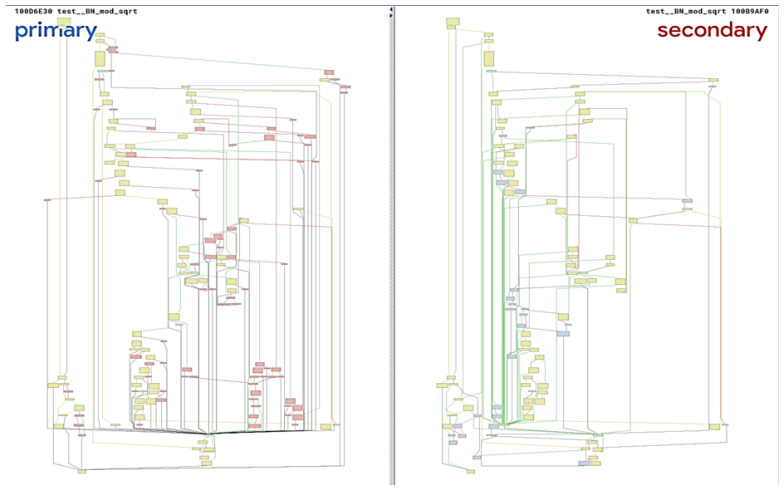
An example of code clones missed by the syntactic analysis.

Existing solutions that compare only the syntactic features of code often miss real clones. The same code, built for different IoT architectures and compilation settings, can differ significantly in syntax. Let us take the widely-known BinDiff utility [24]. Operating at the syntactic level, it builds the CFGs and matches them. For example, Figure 2 shows the CFGs constructed for two instances of the same function compiled for the same device, but for different platforms—ARM and x86. BinDiff detected a significant difference in these code samples, identifying no clone; the similarity score measured by BinDiff is 0.37. These fragments are in fact clones of the same code, performing identical functionality. If this code contained a cloned vulnerability from a software repository, it would bypass this check. That is why code clone detectors should rely on additional high-level information about the code, which introduces a new challenge in clone detection through semantic analysis.

Therefore, it was concluded that syntactic analysis should be reinforced. Modern research suggests two options for such an enhancement:

- The use of multi-static analysis methods at different levels of granularity;
- The simultaneous use of static and dynamic methods.

A combination of several static analysis methods is the most popular approach today, as it does not require additional time or computational overhead. For example, the most popular detectors, like Genius [12] and Gemini [7], employ several code analysis phases. Genius uses a machine learning approach to generate robust, platform-independent function feature vectors, transforming the difference between two functions into a distance between these vectors. Gemini represents the disassembled program code as an attributed CFG (ACFG), where the attributes are a set of metrics calculated during the analysis of the corresponding basic blocks. On a higher level, a special GNN is applied to process ACFGs.

The Asteria utility [25] is based on code decompilation and subsequent construction of an AST, indicating the types of lexemes in the nodes at the pre-processing stage. A Siamese neural network with Tree-LSTM architecture is used as a classifier. An advanced version of this method, Asteria-Pro [26], is specially designed to solve the task of searching for semantic code clones, and it introduces additional steps—preliminary filtering and modifying of the metrics obtained during the comparison based on the analysis of function call graphs.

The BinSlayer method [27] also consists of several code analysis stages. The first one involves applying the BinDiff algorithm, which creates a finite set of matched functions. As a result of this stage, a set of unmatched functions is generated. BinSlayer uses the second stage to find additional comparisons by using the Hungarian algorithm. The graph edit distance (GED) is calculated as a similarity metric for CFG and call graphs. The low-level sources for code feature analysis are the assembler code and the metadata of binary functions, and the high-level features are call graphs and CFGs. The weakness of this method is that the BinDiff output is checked manually by an expert to select really similar code samples.

In [28], the purpose of using low-level features is to reduce the power of a set of unmatched functions before analyzing the high-level features. Filtering is performed based on heuristics: binary functions can be considered clones if the difference in the number of basic blocks and the distance between the vector representations of their normalized assembly code fall below a certain threshold. Following this, it is necessary to check the semantic similarity for each function based on high-level features with a reduced number of candidates. This approach enhances the scalability of the method, enabling it to be applied to large sets of binaries with minimal analysis time. The low-level sources of features in this method are the assembly code and normalized assembly code, while the high-level source is the CFG.

The method proposed in [29] allows searching for borrowed software components in the firmware of IoT devices. This method also uses low-level syntactic features based on the analysis of the assembler code of basic blocks, as well as high-level feature matching. Based on [7], this method also constructs an ACFG and then constructs semantic vectors to determine the similarity of the corresponding code fragments.

In [30], the main stage of binary code analysis is performed based on features generated by in-memory fuzzing. This method just enhances the amount of low-level data for semantic analysis. Low-level features obtained by analyzing assembler instructions complement the semantic signature of each analyzed fragment to improve the result accuracy of classification.

A representative of static-dynamic analysis was proposed in [31]. In the first stage, a static analysis of code was performed, and a CFG was constructed. Secondly, dynamic instrumentation of code was conducted in order to determine the sections of code that were deployed in memory at the execution stage. And, at the third stage, the CFG was modified, referring to data obtained at dynamic analysis. The low-level source of features in this method was the assembly code, while high-level features included information on process behavior and CFG. The use of dynamic analysis and higher-level features helped reduce the impact of software analysis countermeasures on the search for similar code fragments. Unfortunately, this method incurred additional time costs due to the dynamic stage and still operated at the level of common CFGs, which resulted in a loss of valuable information.

Summarizing the comparative survey of the related works and considering the combined methods that support semantic clone detection, Table 1 has been compiled. From the retrospective analysis, the following conclusions can be drawn.

- Methods that offer a sequential comparison of code fragments based on features of code on different granularity levels are more effective than any method that makes a decision based on combinations of code features. This is confirmed by other comparative reviews of methods presented, for example, in studies [32,33];
- Pre-matching and filtering a set of code samples reduces the size of the unmatched set, where semantic methods are applied to identify semantic clones. Such methods utilize machine learning algorithms. Since the unmatched set of code samples, after the syntactic analysis phase, contains only those with structural differences and no syntactic similarity, machine learning models can be tuned more precisely to address the specific task of detecting semantic code clones;
- According to the methods observed, the most efficient and stable results are gained when using graph representations of static and semantic features of code. This leads to the necessity of embedding graphs into low-dimensional vector representations. Intelligent detecting algorithms use GNNs (graph neural networks), RNNs (recurrent neural networks), or CNNs (convolutional neural networks) to produce vectors (e.g., [15,34,35]). According to existing research, GNNs are less time- and memory-intensive on large code bases compared to RNNs and CNNs. But their weakness lies in a high likelihood of collisions, which can result in generating the same attributed vectors for graphs with different topologies and features. Convolutional neural networks tend to treat isomorphic graphs of different functions as similar vectors, while RNN-based methods struggle with functions containing long linear code snippets.

Summarizing the related works, a hypothesis is proposed regarding the need for a novel hybrid syntactic–semantic method to accurately detect semantic clones. It is suggested that syntactic similarity between code samples (e.g., vulnerable code and tested code) be analyzed at the binary code level to optimize overall efficiency and performance. This approach can help reduce the rate of incorrectly matched code samples. At a higher level, the method involves using an attributed AST and a dual deep graph neural network to process semantic features. The specifications of this method and its experimental study are presented in the following subsections.

### 2.2. Code Clone Detection

**Figure 3:**
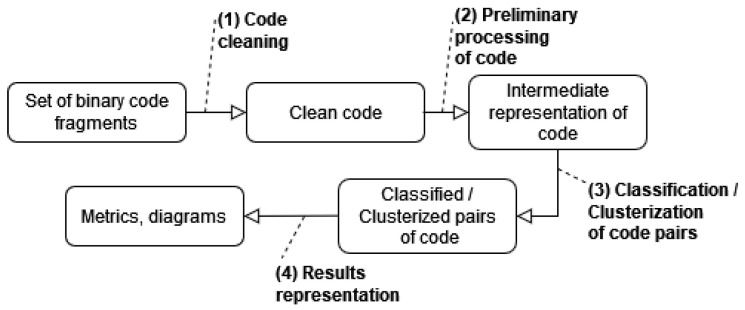
Code clone detection stages.

In a general view, code clone detection can be divided into several typical stages, as shown in Figure 3.

In stage 1, code processing (code cleaning) is performed to remove code sections unused in further analysis, normalize code, and divide large code sections into smaller ones. The input to this stage is unprocessed binaries, and the output is processed binary fragments suitable for further security analysis.

Stage 2 involves a preliminary processing of the code. The comparison algorithms applied later assume the presence of this stage since additional information must be extracted from the code to improve the accuracy of detection. This stage intends to create an intermediate representation of the code fragments and extract their semantic context. Here, methods for code processing may include replacements of instructions and their groups, collecting statistical data on the presence, distribution, and number of instructions of a certain type, building an intermediate representation with formal grammar other than the assembler, recovering the source text, etc. The input to this stage consists of fragments of binary program code, and the output is an intermediate representation of code fragments and/or data obtained from code processing.

In stage 3, code fragments are compared using the data obtained in the previous stage. This stage also involves encoding the data from the preprocessing stage, which is done before comparison. Code features are extracted, and based on this information, a classification task (dividing code fragments into similar or different classes) or a clustering task (grouping code fragments into distinct classes) is performed. The input to this stage includes the intermediate representation of code fragments and/or the information processed in the previous stage.

In stage 4, the results of the code comparison are generated. Methods used at this stage may also output additional data, such as the degree of similarity between samples. It is possible to visualize the results and create specialized diagrams and reports based on user requirements.

The main emphasis is on the preliminary processing of code fragments (stage 2) to obtain the maximum amount of available syntactic data on code features, and on the clone detection stage (stage 3), which performs classification for the accurate evaluation of code semantic similarity.

### 2.3. Preliminary Processing of Code Fragments

**Figure 4:**
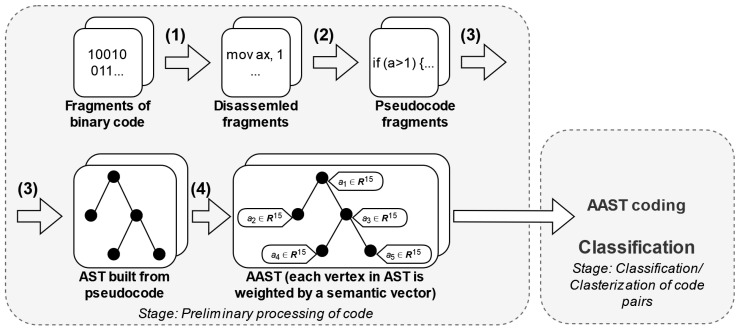
Preliminary processing of the code.

The proposed method for the preliminary processing of binary code fragments includes both the construction of an attributed abstract syntax tree (AAST) and the extraction of semantic features during the analysis of the given code (Figure 4).

The compared code fragments are namely functions. Initially, the functions are disassembled (stage 1 in Figure 4) and decompiled (stage 2). Based on the recovered code, a traditional abstract syntax tree (AST) of each fragment is constructed (stage 3). As a novelty, all nodes of the common AST are proposed to be labeled by semantic attribute vectors (stage 4). The suggested structure is called an attributed AST (AAST).

Each node in the AAST is enriched with a semantic attribute vector that includes key elements, such as the following:

- Semantic representation of the node (i.e., lexeme), obtained by using Word2vec;
- The number of function calls present in the subtree;
- The number of cycles present in the subtree;
- The number of conditional operators present in the subtree;
- The number of switch operators present in the subtree;
- Sum of digital values (values of nodes of int, float types) present in the subtree.

**Figure 5:**
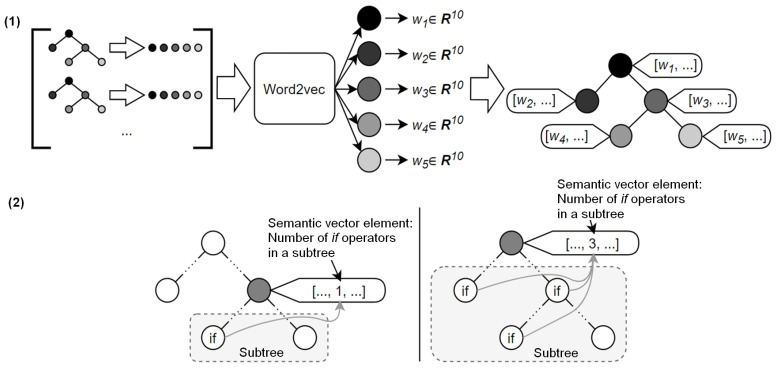
Sample of preliminary processing of binary code fragments.

The scheme for constructing a semantic attribute vector for the AAST node is shown in Figure 5. Figure 5 demonstrates the examples of operator if for a non-terminal node and the root of the tree. In this figure, stage 1 depicts building a Word2vec vector of dimension 10. This stage involves the transformation of the AST into a sequence of node types, training of the model, and mapping each node to its attribute vector as a result of the training. Stage 2 in Figure 5 extends regular AST construction to include the building of elements for the semantic attribute vector of each node, which are computed as the sums of the number of elements of a specific type in the subtree.

The Word2vec semantic vector, associated with each node in the AAST attribute vector, allows us to estimate the distance between the lexemes in a language. In this sense, the formal grammar of the pseudocode can be considered an analog of natural language. The distance between lexemes that are most often found in adjacent positions is small. Conversely, lexemes that cannot be located next to each other in the program code are associated with Word2vec vectors, which have a large distance between them. Other elements in the semantic vector for a node are calculated as the sum of the occurrences of lexemes of a specific type within a subtree. Thus, when a modification is made to the analyzed code (e.g., closing a vulnerability), the change in the code structure propagates to the root and the subtrees containing the modified nodes.

### 2.4. Stage of Classification

**Figure 6:**
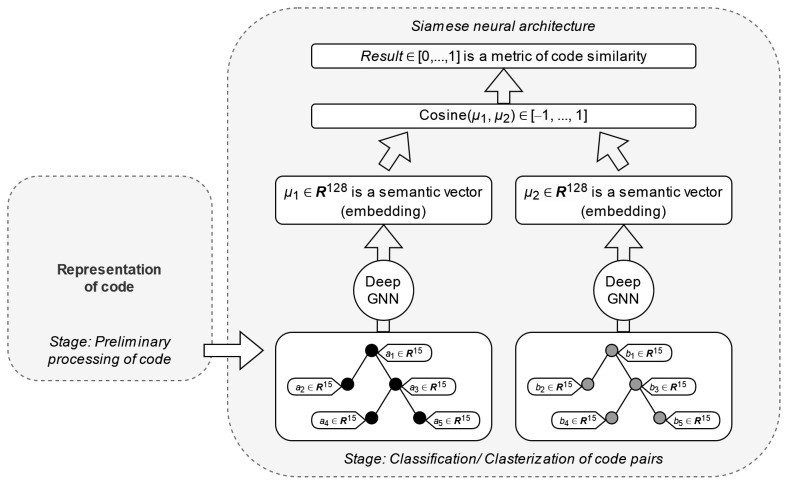
Code clone detection.

The method considered in [7] is chosen to implement the classification stage. Each AAST obtained in the preliminary processing stage is encoded into a semantic vector using a graph neural network (GNN) via the structure2vec vector representation method [32]. The original GNN model is replaced with a deep learning GNN (deep GNN) with three neural layers. Two instances of deep GNN are combined into a Siamese neural network. This allows the neural network to be trained to generate semantic vectors and compare pairs of vectors in every training epoch, implementing supervised learning. The diagram of the designed method is shown in Figure 6.

In Figure 6, the neural network is fed with a pair of AAST instances corresponding to code clones (or different code samples), e.g., a code sample and a vulnerability sample. The deep GNN mapping in each branch converts the input instance into a vector representation μ (stage 1 in Figure 6). To compare the resulting vectors, the cosine distance is calculated (stage 2):

$$cosine\left({\bar{\mu }}_{1},{\bar{\mu }}_{2}\right)=\;\frac{{\bar{\mu }}_{1}\cdot {\bar{\mu }}_{2}}{∥{\bar{\mu }}_{1}∥∥{\bar{\mu }}_{2}∥}=\frac{\sum_{i=1}^{n}\mu_{1i}\mu_{2i}}{\sqrt{\sum_{i=1}^{n}\mu_{1i}^{2}}\sqrt{\sum_{i=1}^{n}\mu_{2i}^{2}}},$$

where ${\bar{\mu }}_{1}$ and ${\bar{\mu }}_{2}$ are semantic vectors obtained as the results of the GNN, and $\mu_{1i}$ and $\mu_{2i}$ are the *i*-th components of the vectors $\mu_{1}$ and $\mu_{2}$, respectively.

Stage 3 in Figure 6 is the normalization of the result of the comparison.

The mapping is implemented using a fully connected neural network, onto which a mask corresponding to the topology of the next AST is imposed at each step [7]. The use of this type of neural network allows the influence of the graph vertex attribute (semantic context) to be extended to the vertices incident to it. This ensures compliance with the tree topology. For each AST vertex, its own embedding is constructed, which is then summed by every instance. The embedding μ of the vertex *v* at step T+1 is specified by the following formulas:

$$\mu_{v}^{\left(T+1\right)}=L\left(x_{\nu },\sum_{u\in N\left(\nu \right)}\mu_{u}^{\left(T\right)}\right),\;\forall v\in V;$$

$$L\left(x_{\nu },\sum_{u\in N\left(\nu \right)}\mu_{u}\right)=tanh\left(W_{1}x_{\nu }+\sigma \left(\sum_{u\in N\left(\nu \right)}\mu_{u}\right)\right);$$

$$\sigma \left(a\right)=P_{1}\times ReLU\left(P_{2}\times \ldots ReLU\left(P_{n}a\right)\right),$$

where $x_{\nu }$ denotes a vector of attributes of the vertex dimension *d* (vertex context), L denotes nonlinear mapping; $N\left(\nu \right)$ denotes a set of vertices incident to the given one; $W_{1}$ denotes a matrix of size d×p; $P_{1}$ denotes a matrix of size p×p; and *p* denotes the embedding dimension.

**Figure 7:**
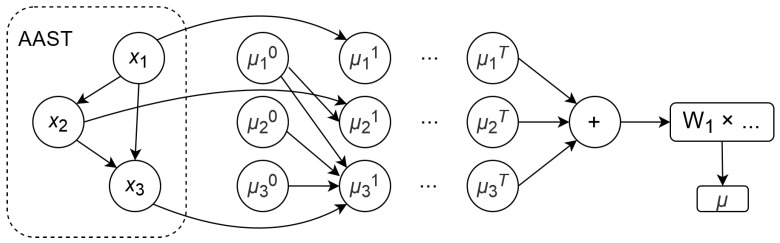
Neural network configuration.

The operation diagram of a fully connected neural network in each branch is presented in Figure 7.

The minimal Euclidean distance between the similarity label of a code pair and the output obtained during training is chosen as the optimization goal. The hyperparameters for training the Siamese neural network were selected experimentally, following [7]. The best results were achieved when training the model with three layers in fully connected subnetworks, 100 epochs, and five context propagation iterations. The dimension of the semantic vector for each compared code fragment is 128.

### 2.5. Combination of Syntactic and Semantic Analyses

As noted above, code clones can be detected in terms of code similarity. Methods for determining the similarity of binary code fragments can be based on the analysis of syntactic and semantic features.

Commonly used syntactic features are as follows:

- Byte sequences;
- Assembler instruction sequences;
- Statistical values extracted from the analysis of byte and instruction sequences.

In methods for syntactic detection of code clones, sequences are used as code features, forming the basis for hash matching, machine learning, and comparisons of PDG and CFG. Thus, the granularity level of syntactic similarity is close to the byte level due to the syntactic features.

To improve detection accuracy and address differences in the compilation parameters and architecture, clone detectors should rely more heavily on semantic information around the code. Semantic features describe the relationships between code fragments and are derived from the analysis of intermediate and vector representations of the code. Extracting these features requires analysis at higher granularity levels. Determining the optimal set of granularity levels and sources for features at each level presents a novel challenge for code clone detection that has already begun to be explored in related studies (e.g., [33,34,35,36,37]).

Data source choices for creating code semantic features affect the efficiency of code clone detectors. The use of only low-level syntactic features based on binary and assembler analysis leads to poor classification performance. Insufficient knowledge about the semantics of the code makes it difficult to analyze, as data sources related to the code structure, input and output parameters, external functions used, and symbol information are not involved in clone detection [28]. On the other hand, using only high-level features (e.g., graph or vector representations) in semantic clone detection methods often results in a high number of false positives (i.e., non-clone samples being incorrectly identified as clones).

When combining high-level and low-level features within a single method, there is a tendency to reduce the efficiency of syntactic clone detection, which is expressed in an increased rate of false positives and false negatives [34]. This problem degrades the quality of the proposed method. Our method is based on the analysis of AAST. It is constructed from binary code fragments and uses the recovered program code. Sources of code features are disassembled code, recovered code, and graph representation. In this case, a relatively high false negative rate is expected. This leads to the omission of similar code fragments. In practice, this is the most critical issue, as it affects the usefulness of the proposed method. A false positive rate is less critical—it slightly increases the number of code fragments that require expert assessment for similarity. To address this issue, we propose a sequential extraction and use of both low-level and high-level features. Additionally, we suggest evaluating the potential of combining semantic features extracted from the AAST with syntactic and structural code features.

To enhance our original method, the BinDiff method [24] was involved to determine the syntactic similarity of code fragments. BinDiff implements the following stages:

- Initial matching involves matching function signatures, which include the number of basic blocks, the number of edges in the CFG, and statistical data on the number of specific instruction types within functions. At this stage, the call graph is also matched, which is constructed for each analyzed code sample.
- Attribute-driven similarity determination: The similarity of functions successfully matched in the previous step is evaluated using key attributes. These attributes include the hash of the function name, the hash of the function body, the matching of function positions within the call graph, etc.
- For matched functions, their CFGs are compared to detect modifications at the level of individual instructions.

A formal description of the combined method is presented as follows:

$B_{1}$ and $B_{2}$ are the sets of binary functions of the first and second code samples, respectively. *M* denotes the set of pairs of functions obtained as a result of the bijective mappings $M_{1}\to M_{2}$, where $M_{1}$ and $M_{2}$ are sets of matched functions in the first and second code samples, respectively; $M_{1}\subseteq B_{1}$, $M_{2}\subseteq B_{2}$.

$U_{1}$ and $U_{2}$ are sets of unmatched functions in the first and second code samples, respectively; $U_{1}=B_{1}\setminus M_{1}$, $U_{2}=B_{2}\setminus M_{2}$. Then, the result of the syntactic analysis can be described as $M,U_{1},U_{2}=BinDiff\left(B_{1},B_{2}\right)$, where $BinDiff(B_{1},B_{2})$ denotes a function implementing the BinDiff method for a syntactic comparison of code samples.

Now, let $M^{\prime }$ denote the set of pairs of functions obtained as a result of the mapping ${M_{1}}^{\prime }\to {M_{2}}^{\prime }$, where ${M_{1}}^{\prime }$ and ${M_{2}}^{\prime }$ are the sets of mapped functions in the first and second code samples, respectively; ${M_{1}}^{\prime }=B_{1}$, ${M_{2}}^{\prime }\subseteq B_{2}$. Moreover, $\forall m_{1}\in M_{1}\;\exists \left\{{m_{2,i}}^{\prime }\right\}$ there exists a set of candidates sorted in descending order of the similarity metric. Then, the results of the semantic analysis are specified as $M^{\prime },{U_{2}}^{\prime }=SemCom\left(B_{1},B_{2}\right)$, where $SemCom(B_{1},B_{2})$ denotes the function implementing the semantic comparison of code samples based on the intelligent AAST-based method presented in Section 2.4.

Now, the algorithm for operation at the preliminary stage can be formally presented as Algorithm 1.
**Algorithm 1** Algorithm for the preliminary stage.**Input:** $B_{1},B_{2}$**Output:** *M*, $M^{\prime }$      1:$M,U_{1},U_{2}=BinDiff\left(B_{1},B_{2}\right)$      2:$M^{\prime },{U_{2}}^{\prime }=SimCom\left(B_{1},B_{2}\right)$      3:**return** $M,M^{\prime }$     ▹ Save them to the base of code matching

Let $search_{BinDiff}\left(m_{1}\right)=V$, $m_{1}\in M_{1}$, V=∅, or $V=\{m_{2}\}$, $m_{2}\in M_{2}$ be a function returning the image $m_{1}$ of the mapping to $M_{2}$, obtained at the preliminary stage. $sim_{BinDiff}\left(m_{1},m_{2}\right)$ denotes the similarity metric of binary functions $m_{1}$ and $m_{2}$.

Let $sim_{SimCom}\left({m_{1}}^{\prime },{m_{2}}^{\prime }\right)$ be a similarity metric obtained by semantic analysis. A function that returns an image of the mapping ${m_{1}}^{\prime }$ to ${M_{1}}^{\prime }$ is $search_{SimCom}\left({m_{1}}^{\prime }\right)=\left\{{m_{2,i}}^{\prime }:sim_{SimCom}\left({m_{1}}^{\prime },{m_{2,i}}^{\prime }\right)>sim_{SimCom}\left({m_{1}}^{\prime },{m_{2,i+1}}^{\prime }\right)\right\}=V^{\prime }$.

Therefore, the classification stage can be presented as Algorithm 2. Since ${M_{1}}^{\prime }=B_{1}$, this algorithm returns a set of clones for a query $m_{1}$.
**Algorithm 2** Algorithm for the classification stage.**Input:** *M*, $M^{\prime }$, and $m_{1}$ denotes a query function**Output:** *V* or $V^{\prime }$      1:**if** $sim_{BinDiff}\left(m_{1},m_{2}\right)>k$ **then**      2:    **return** *V*        ▹*k* denotes a threshold value of the similarity metric      3:**end if**      4:**if** V≠∅ AND $V\subseteq V^{\prime }$ **then**      5:    **return** *V*      6:**else**      7:    **return** $V^{\prime }$      8:**end if**

Syntactic features extracted during code analysis are assembler instructions, including execution flow branching instructions, which allow searching for isomorphisms of CFGs and matching the corresponding basic blocks. Semantic features are vector representations that are built based on the AAST of the reconstructed code of each analyzed sample using a deep GNN based on the structure2vec method [32], as described above in Section 2.4. The output of this method includes a list of code fragments that are most likely clones of the given code.

**Figure 8:**
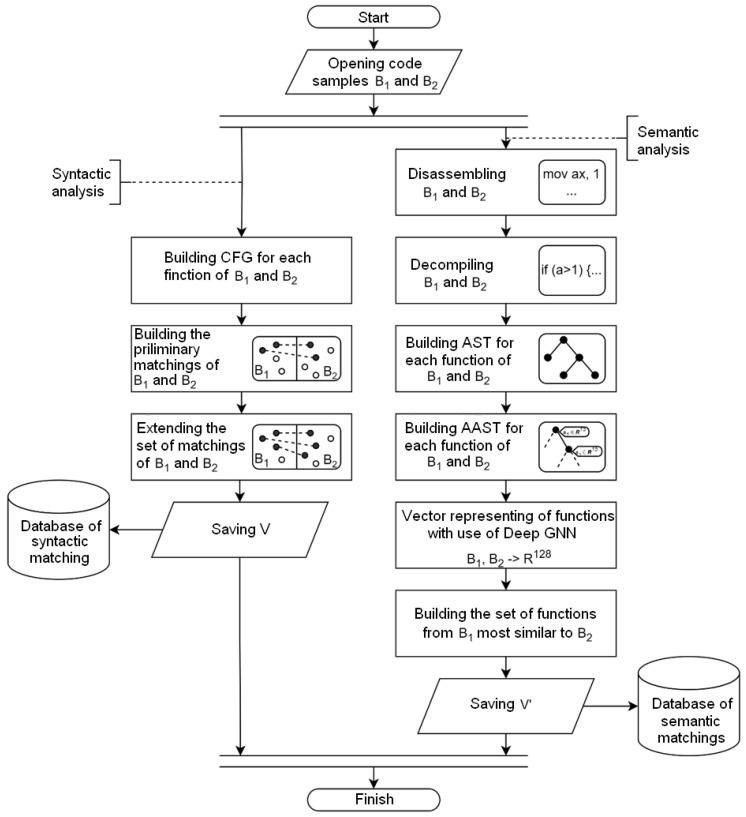
The proposed syntactic–semantic method.

A flow chart for the proposed syntactic–semantic method is presented in Figure 8.

The proposed method focuses on searching for code clones by combining the syntactic and semantic code features. Since the set of syntactic clones is a subset of semantic clones, syntactic analysis allows us to detect syntactic clones and retrain a neural network for detecting semantic clones with minimal structural and syntactic similarity. To speed up the clone search, this method can be assisted by an adjustable repository of features extracted from code samples during analysis.

### 2.6. A Demonstration Example

For a better understanding of the work of the proposed syntactic–semantic method, the demonstration example is presented as follows.

**Figure 9:**
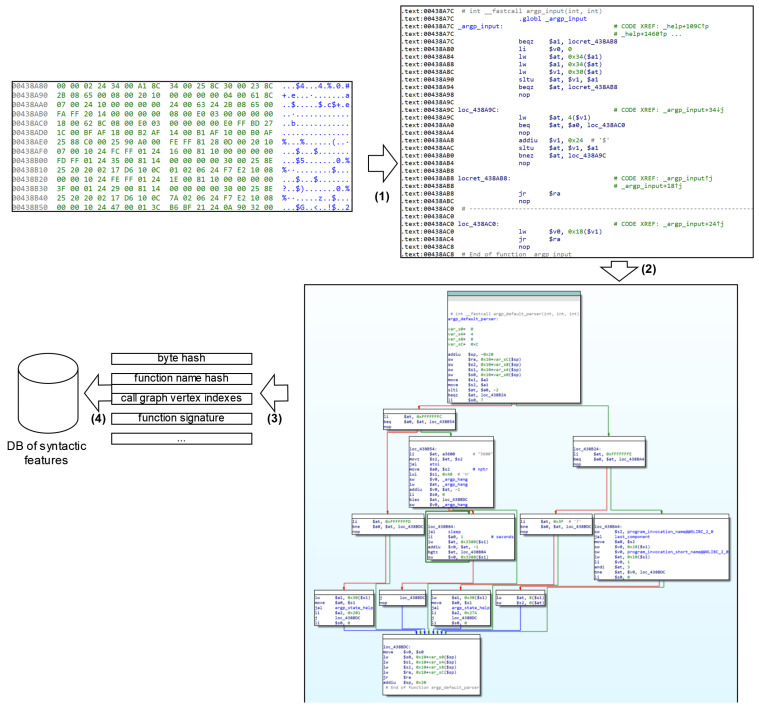
Syntactic feature extraction in the preliminary stage.

Figure 9 shows an example of code feature processing during syntactic feature extraction in the preliminary stage. In stage 1, syntactic feature extraction is performed using a BinDiff-inspired algorithm. The code is decompiled and CFG is built (stage 2). In stage 2, the extracted attribute tuple and associated function name are stored in the syntactic attributes database for further comparison (stage 4).

**Figure 10:**
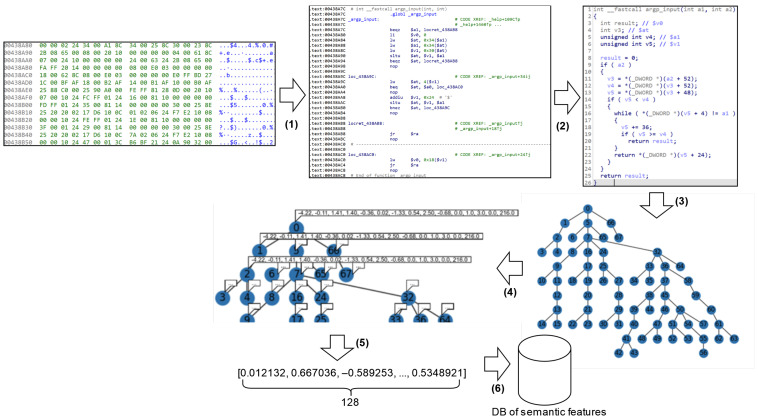
Semantic feature extraction in the preliminary stage.

Figure 10 shows an example of feature processing during semantic feature extraction in the preliminary stage. In stage 1, the code of the function under investigation is disassembled. In stage 2, the decompiled code is generated. Based on the obtained C-like code, the abstract syntactic code of the function (stage 3) is constructed. In stage 4, each node of the AST is provided with attributes: part of the vector represents statistical attributes and part of the vector is a Word2vec semantic vector. In stage 5, the obtained AAST is converted into a vector representation (embedding) using a GNN. Finally, the obtained vector representation and associated feature name are stored in the semantic features database for further comparison (stage 6).

**Figure 11:**
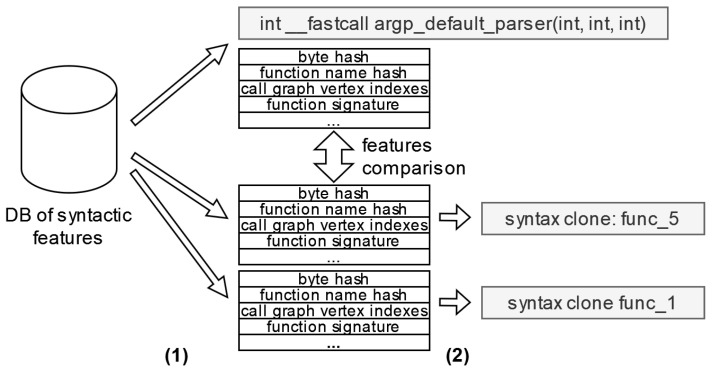
Syntactic features comparison.

Figure 11 shows an example of detecting syntactic clones. From the syntactic attributes database, tuples of attributes are extracted, with one tuple corresponding to the query function (stage 1). The attributes of the query function are then sequentially compared with the corresponding attributes of other functions included in the comparison. Attributes are prioritized during the comparison process and are assigned a weight that influences the comparison result. The functions with the highest similarity scores form a list of functions that are syntactically similar to the query function (stage 2).

**Figure 12:**
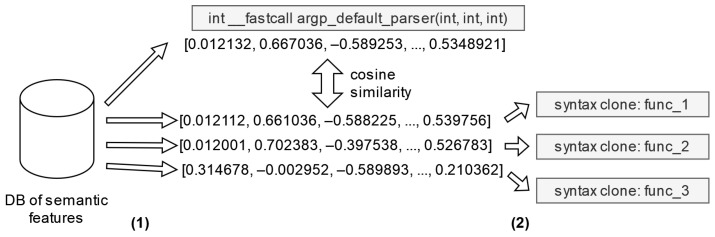
Comparison of semantic features.

Figure 12 shows an example of detecting semantic clones. Embeddings are extracted from the semantic features database, one of which corresponds to the query function (stage 1 in Figure 12). Using the cosine distance metric, the vectors that are least distant from the given feature are determined. The features to which such vectors correspond form the list of features semantically similar to the original one (stage 2). Since the set of syntactic clones is not a subset of the set of semantic clones, the set of semantic clones is returned as the result, as specified in Algorithm 2.

## 3. Results

In an experimental study, the proposed method was tested for its efficiency. It was compared to popular BinDiff, Gemini, and Asteria detecting tools. A prototyping utility that implements the proposed method was implemented in Python. Incode functions were accepted as input code fragments. The sample function code was determined using the IDA Pro 7.7 toolkit. The preprocessing module receives a text representation of the decompiled code from IDA Pro. The module’s output consists of files containing data on code functions in JSON format. Next, the classification module receives a set of AAST functions during training, with the information saved in JSON format. The output of the module is a similarity metric for the code samples.

The following actions are performed during the preprocessing stage:

1. Disassembling and restoring the function code using IDA Pro 7.7.
2. Building an AST for the restored code of all functions.
3. Training the Word2vec model on the combined set of lexeme types of all executable files. Sequences of lexemes of function bodies are used as sentences (continuous sequences of tokens). A mapping of the lexeme set onto a set of semantic vectors is formed.
4. Each node is assigned an attribute vector consisting of a Word2vec semantic vector and statistical information on the number of lexemes of a certain type in a subtree. As a result, the AASTs are built.

Before training, the built AASTs are clustered by name: functions with the same name obtained from different binary files are considered to be semantic clones. Within each epoch, training is performed on groups of function pairs. Each group contains five pairs of semantically different functions and five pairs of clones. Pairs of different functions are formed randomly, while pairs of similar functions are taken from the same equivalence class (cluster). Training continues for 100 epochs.

To train and test the solution, a dataset based on Linux utilities was used. Binary files were compiled with different optimization parameters, \O0 and \O2. The set of functions in the dataset was divided into training, validation, and testing subsets in an 80:10:10% ratio. Statistics on the datasets are listed in Table 2.

After training the developed utility, the detection quality was tested. For this purpose, representatives of IoT software were taken as binary files (Table 3).

The first experiment was an ablation study aimed at validating the necessity of each component of the proposed method. While a hybrid approach combining syntactic and semantic analyses was presented, it may not have been clear whether each step of the method is essential for achieving the reported goals. An ablation study involves systematically altering parts of the method and observing the impact on the results. To do this, our method was compared with the BinDiff utility, a state-of-the-art tool for clone searching that implements syntactic-only analysis. Next, we disabled the syntactic component of our method and tested it in semantic-only mode. And, finally, our method was run in full mode, involving both syntactic and semantic parts at once.

**Figure 13:**
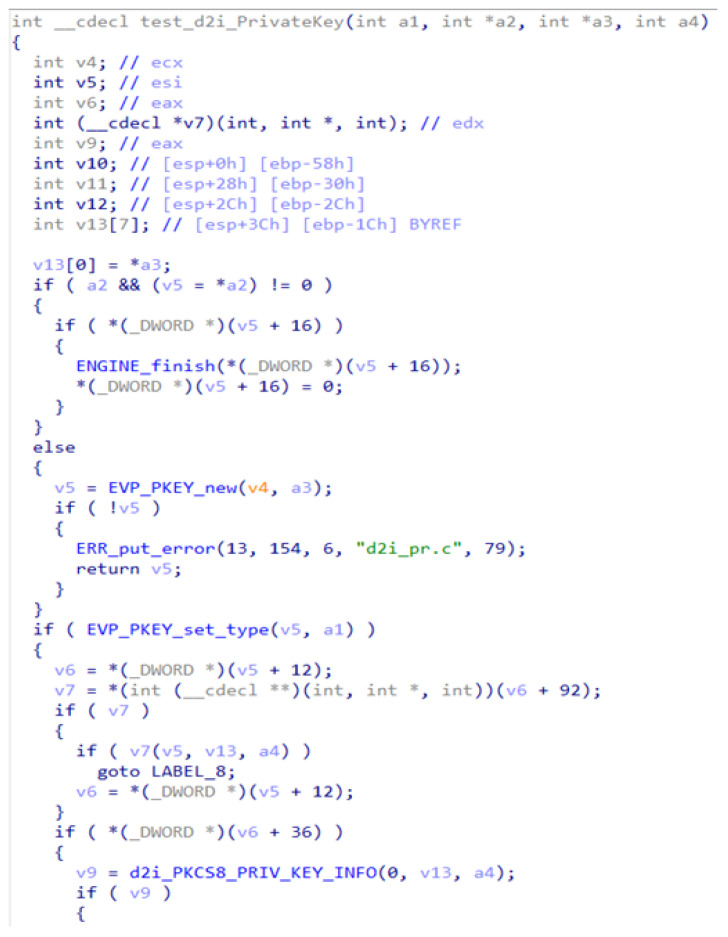
A sample function implemented in the IoT platform.

**Figure 14:**
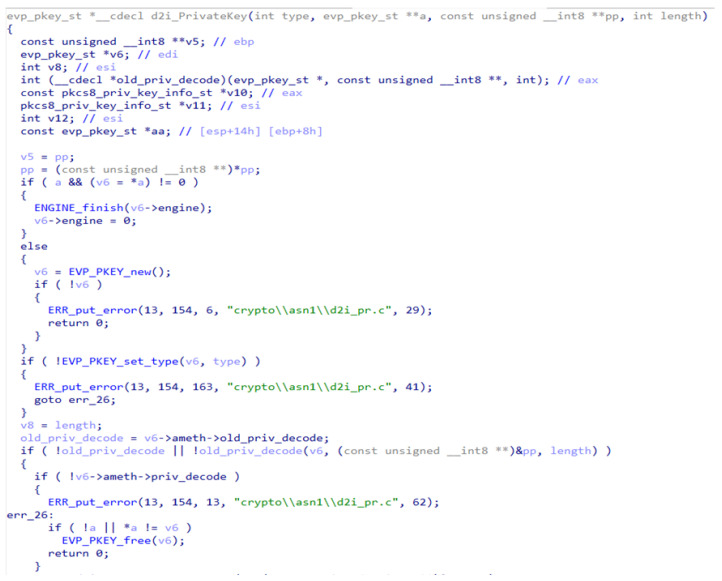
The same function as in Figure 13, but implemented in the PC platform.

For the ablation study, test input was taken from the binaries presented in Table 3. For example, samples of the restored code for one function taken from different executable files are presented in Figure 13 (taken from libcrypto.so.1.0.0, developed for the IoT platform on a Linux system with MIPS architecture) and Figure 14 (taken from libcrypto-1_1.dll, developed for the PC platform on an MS Windows system with x86 architecture). It is a single function that performs the same operation but has a different code representation due to code reuse. For more efficient software production, this function implementation is cloned from PC implementation of the library to IoT implementation of the same library. In the same manner, clone-caused vulnerabilities are transferred from one software type to another. The test aimed to evaluate the detection of semantic clones using different variants of syntactic and semantic analyses and to determine whether the IoT code was cloned.

**Figure 15:**
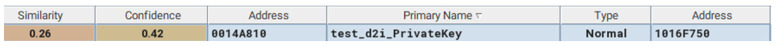
A sample of the BinDiff output.

**Figure 16:**
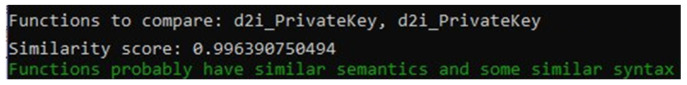
A sample of our tool output.

The BinDiff utility (syntactic-only analysis) demonstrated the output as presented in the screenshot in Figure 15. BinDiff showed a similarity score of 0.26, i.e., BinDiff did not detect the cloned code. The output of our method (syntactic–semantic analysis) is presented in Figure 16—the proposed method showed a similarity score of 0.996. To completely demonstrate the usefulness of a syntactic–semantic symbiosis, the semantic part of our method stayed active while the syntactic one was switched off. The syntactic-only case showed a similarity score of 0.89. All scores obtained for different syntactic and semantic combinations are summarized in Table 4. This ablation study provides compelling evidence that the syntactic–semantic approach is indeed effective.

In the second stage of experiments, the concurrent popular solutions were tested by comparing the efficiency with the proposed syntactic–semantic method. The choice of candidate methods for code clone detection was based on the high efficiency reported by their developers (Asteria [25] and Asteria-Pro [26]) and their stable performance across different code bases (Gemini [7]).

Gemini [7] uses a GNN to generate a vector representation of the CFG added with numerical statistics. This is one of the first and most effective methods to use a machine learning approach to compare code samples based on attributed CFGs. The Asteria utility [25] is based on code decompilation and subsequent construction of ASTs, indicating the types of lexemes in the nodes at the preprocessing stage. A Siamese neural network with Tree-LSTM architecture is then applied as a classifier. Asteria-Pro [26] introduces additional steps: preliminary filtering and analysis of the CG metrics.

**Figure 17:**
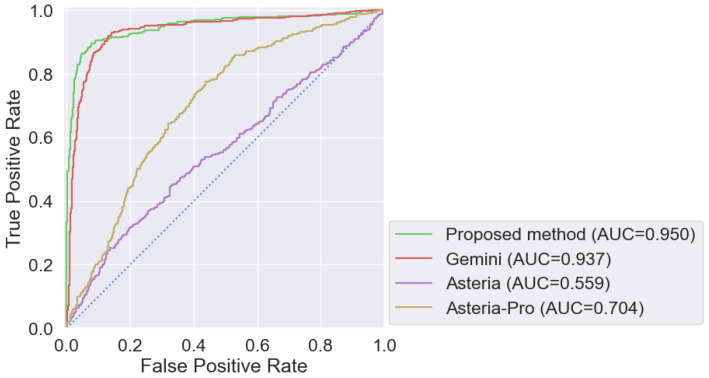
Comparison of ROC and AUC values.

The implementations of these methods were tested using the same dataset (Table 2). For Gemini, the model was trained using the parameters proposed by the authors in [38]. For Asteria and Asteria-Pro utilities, a trained model proposed by the authors in [39] was applied. The ROC (receiver operating characteristic) curves were plotted as a result of this comparative experiment. The obtained ROC curves and the corresponding areas under the ROC curves (AUC scores) are presented in Figure 17. Values for quality metrics (the commonly used recall, precision, and F1 metrics) collected during the experiment are presented in Table 5.

The experimental results show that the syntactic–semantic method demonstrates superior performance in semantic clone detection compared to syntactic-only, semantic-only, and other popular solutions.

## 4. Discussion

The proposed syntactic–semantic method has shown the best detection quality with the least amount of errors. For example, in experiments, Gemini showed good results, but results that were lower than our method, indicating consistently high efficiency when applied to different data. The indicators for Asteria and Asteria-Pro utilities showed that when identifying similar code samples based on AST, relying only on the tree structure is not sufficient. To improve Asteria’s quality, it is necessary to extract additional semantic information and include it directly in classification, not after comparison.

Our method, presented in this paper, differs from other combined methods, not only in the analysis of CG, CFG, and assembler instructions but also in the suggested analysis of the attributed AST structures during the semantic analysis of code. This allows our method to effectively identify and eliminate syntactic clones from further consideration. This has a positive effect on performance and the ability to apply the method to large sets of binary codes. Due to the preprocessing stage and preliminary syntactic analysis of all the code bases, we exclude syntactic code clones and reduce the code base. This allows us to avoid most collisions when using GNNs to produce embeddings. This also leads to better detection quality compared to concurrent solutions.

As shown in Table 4, combining syntactic and semantic analyses for software clone detection improved the efficiency of clone detection. The method of syntactic similarity detection showed low efficiency when comparing functions from executable files designed for different platforms. As previously noted, this is due to significant changes in the CFG and CG of the compared functions during the compilation of executable files for different platforms, making it challenging to detect vulnerabilities using borrowed code detection methods in IoT platforms.

Using only the semantic clone detection method proposed in this paper reduces the number of FNs but increases the number of FPs, resulting in a successful comparison of functions that are not semantic clones. This issue arises due to similarities in the AST structure and vertex attributes.

The proposed syntactic–semantic method reduces the problems encountered when using syntactic and semantic fragment comparison methods separately. By applying the syntactic analysis part, it is possible to exclude pairs of features that are highly likely to be clones. This reduces the number of functions with similar AST structures and vertex attributes, which consequently reduces the number of errors in determining semantic clones.

Concerning the method’s weaknesses, due to its architecture, the proposed method does not perform a targeted search for clone-caused vulnerabilities of specific types. Potentially malicious code fragments are identified by comparing them with known (semantic) patterns. That is why it is impossible to search for an unknown vulnerability or exploit with unknown semantics by using the proposed method. Nevertheless, this limitation can be eliminated by introducing an additional stage of vulnerability detection through the dynamic analysis of software behavior.

To complete the discussion, Table 1 presented in Section 2.1 is extended by the string corresponding to the proposed method.

## 5. Conclusions

Concerning the IoT ecosystem’s security, our research analyzes methods for detecting clone-caused vulnerabilities using code clone searching based on both syntactic and semantic features. Following the survey of related code clone detection methods, we hypothesized that a hybrid syntactic–semantic approach is needed for the effective detection of any of the four possible clone types.

A hybrid syntactic–semantic detecting method for code clone detection is proposed and developed. The syntactic similarity between code samples (e.g., vulnerable code and tested code) is analyzed at the binary code level to optimize efficiency and performance. The semantic part of the proposed method is based on the analysis of attribute abstract syntax trees by using double deep graph neural networks. Based on this, it became possible to reduce the rate of incorrectly matched code samples. The developed method demonstrates better accuracy (AUC = 0.962), with the least amount of errors than the popular competitors.

Our future research will focus on analyzing the potential of applying the proposed method to automatically search for clone-caused vulnerabilities using both semantic and syntactic signatures. We will develop advanced methods to automatically create training samples using machine learning algorithms for determining code similarity. We also plan to explore methods based on combining static and dynamic analysis techniques to detect unknown vulnerabilities.

## Figures and Tables

**Table 1:** The combined methods for code clone detection.

Method	Low-Level Features	High-Level Features	Detecting Technique	Combining Technique	Specifics
Genius [12]	Assembler code	CFG	Static. CFG + functional vector distance calculating	Sequential application to reduce the power of multiple unmatched samples	Analysis of the CFG alone does not provide enough semantic information to determine similarity accurately.
Gemini [7]	Assembler code	ACFG	Static. ACFG + GNN	Sequential application to reduce the power of multiple unmatched samples	Analysis of the CFG only does not provide enough semantic information to determine similarity accurately.
Asteria [25], Asteria-Pro [26]	Assembler code, function metadata	ACFG, AST, code statistics	Static. ACFG + AST + GNN + Tree-LSTM	Sequential application to reduce the power of multiple unmatched samples	Many special sequential steps for data processing. The method requires a lot of time. The results are not high, because collected semantic information is not comprehensive.
BinSlayer [27]	Assembler code, function metadata	CG, CFG	Static. BinDiff + Hungarian algorithm for matching functions by GED	Sequential application to reduce the power of multiple unmatched samples	It can be applied to large sets of code samples. Matched samples are excluded before applying the Hungarian algorithm.
BinSequence [28]	Assembler code, normalized assembler code	CFG	Static. Preliminary analysis of similarity of the number of basic blocks, the vector representations of normalized assembler code + Analysis of similarity of paths in CFG	Sequential application to reduce the power of multiple unmatched samples	It can be applied to large sets of code samples. Clone detection is performed using graph theory only, without machine learning.
Zhao et.al. [29]	Assembler code	ACFG	Static. ACFG analysis + GNN	Code features are combined within a single method to produce a decision on the similarity	Analysis algorithm is difficult to scale because there is no preliminary reduction in the set power.
IMF-SIM [30]	Assembler code	Process execution traces	Static + Dynamic. Reverse taint-analysis to resolve data types + Construction and comparison of program execution traces based on in-memory fuzzing	Code features are used sequentially and cyclically	High complexity. It requires a secure execution environment for the software being analyzed. It also requires a lot of time for high code coverage.
Roundy et.al. [31]	Assembler code	CFG, process behavior	Static + Dynamic. Analysis of CFG isomorphisms + Modifications of CFG based on data from code execution with instrumentation	Sources of code features are used sequentially and cyclically: construction of CFG based on static analysis, obtaining data from dynamic analysis, modification of CFG, etc.)	High complexity: it requires a secure execution environment for the software being analyzed. Analysis of the CFG alone does not provide enough semantic information to determine similarity accurately.
Proposed method ^1^	Assembler code, function metadata	CG, CFG, AAST	Static. BinDiff + AAST + two deep GNNs	Sequential application to reduce the power of the set of multiple unmatched samples	It can be applied to large sets of code fragments. BinDiff output is refined using comprehensive machine learning analysis of AAST. Modular (e.g., BinDiff can be replaced with another extraction algorithm).

^1^ The proposed method is included here to provide a complete comparison with other methods.

**Table 2:** Datasets used for the experimental study.

Dataset	Num. of Functions in Dataset	Clusters	Num. of Clusters in Dataset
Training dataset	8267	In training dataset	3416
Validation dataset	1116	In validation dataset	486
Testing dataset	1276	In testing dataset	474
Total	10,659	Total	4376

**Table 3:** Binaries used for the experimental study.

Binary File	Software	System	Architecture	Compiled with Optimization
libcrypto.so.1.0.0	OpenSSL v. 1.0.0, open source library (OpenSSL Software Foundation Inc., Newark, DE, USA)	Linux	MIPS	\O2
libcrypto-1_1.dll	OpenSSL v. 1.1.1, open source library (OpenSSL Software Foundation Inc., Newark, DE, USA)	Windows	x86	\O2

**Table 4:** Efficiency of different syntactic and semantic combinations.

Characteristic	Syntactic-Only (BinDiff Works)	Semantic-Only (Only Semantic Part of the Proposed Method Works)	Syntactic–Semantic (Proposed Method Works)
Similarity score	0.26	0.89	0.996

**Table 5:** Experimental results.

Method	Recall	Precision	F1
Gemini	0.880	0.889	0.884
Asteria	0.510	0.554	0.531
Asteria-Pro	0.698	0.648	0.672
Proposed method	0.907	0.894	0.900

## Data Availability

Data are contained within the article.
